# Factors Influencing Medication Non-Adherence among Chinese Older Adults with Diabetes Mellitus

**DOI:** 10.3390/ijerph17176012

**Published:** 2020-08-19

**Authors:** Ningze Xu, Shiyu Xie, Yingyao Chen, Jiajia Li, Long Sun

**Affiliations:** 1 School of Public Health, Fudan University, Shanghai 200032, China; nzxu17@fudan.edu.cn (N.X.); syxie14@fudan.edu.cn (S.X.); 2 Key Lab of Health Technology Assessment, National Health Commission of the People’s Republic of China (Fudan University), Shanghai 200032, China; 3 School of Public Health, Shandong University, Jinan 250012, China; lijiajia@sdu.edu.cn (J.L.); sunlong@sdu.edu.cn (L.S.); 4 Collaborative Innovation Center of Social Risks Governance in Health, School of Public Health, Fudan University, Shanghai 200032, China

**Keywords:** medication non-adherence, diabetes mellitus, elderly, cross-sectional study, China

## Abstract

Objectives: This study aimed to examine the prevalence of medication non-adherence among older adults with diabetes mellitus (DM) in Shandong province, China and to identify its influencing factors. Methods: A sample of 1002 older adults aged 60 or above with DM was analyzed. Medication adherence was measured using the Morisky–Green–Levine (MGL) Medication Adherence Scale. Descriptive statistical analysis, chi-square test, univariate and multivariate logistic regression analyses were employed. Results: The prevalence of self-reported medication non-adherence among older adults with DM was 19.9%. Female respondents (adjusted odds ratio (AOR) = 1.56, 95% CI: 1.09–2.24) and respondents who perceived medication adherence to be unimportant (AOR = 1.69, 95% CI: 1.05–2.74) were more likely to experience medication non-adherence. Respondents with 5 years of disease duration or longer were less likely (AOR = 0.63, 95% CI: 0.46–0.87) to experience medication non-adherence. Conclusions: This study showed that about one out of five older adults with DM in Shandong province, China, experienced medication non-adherence, and that gender, disease duration and perceived importance of medication adherence were associated with medication non-adherence in this population group. Provision of counseling and health education programs could be the future priority to raise patients’ awareness of the importance of medication adherence and improve patients’ self-management of DM.

‘Drugs don’t work in patients who don’t take them’.

[1]—C. E. Koop, M.D.

## 1. Introduction

Characterized by hyperglycemia, diabetes mellitus (DM) is a group of chronic, metabolic diseases in which the pancreas does not secrete enough insulin, a hormone regulating blood sugar, and/or the body develops resistance to insulin it produces [2,3]. As the disease progresses, patients are at risks of experiencing damages to the heart, blood vessels, kidneys, eyes, feet and/or nerves [4]. With population aging, rapidly increasing urbanization, expanding obesogenic environment and other prevailing risk factors, the prevalence of DM has been on a steady rise during the past decades, which renders DM a major public health challenge in the world [4,5]. According to the global estimates released by the International Diabetes Federation (IDF), there were 463 million adults with DM in 2019, and this figure, if no countervailing measures are taken, will escalate to 578 million in 2030 and even to 700 million in 2045 with a 51% increase [5]. It is also noteworthy that the prevalence of DM increased with age, and it is estimated that the global prevalence was 19.9% (111.2 million people) in adults aged 65–79 years in 2019 [5]. In addition to high prevalence, DM directly caused 1.6 million deaths worldwide and ranked eighth among the leading causes of death worldwide in 2016 [4,6]. To relieve the heavy burden of disease directly and indirectly caused by DM, achieving the targets for glycemic control among patients with DM is crucial [7,8]. Adequate glycemic control is important in preventing or delaying the onset of complications related with DM, and medication adherence is one of the top determinants to achieve this goal [4].

According to the commonly used definition, medication adherence refers to ‘the extent to which patients take medications as prescribed by their healthcare providers’ [9]. Medication non-adherence is a prevalent phenomenon; even in the settings of clinical trials where patients were rigorously selected and received close attention to take medications, average medication non-adherence rates among patients with chronic conditions still range from 22% to 57% [9,10]. Medication non-adherence also brings significant preventable costs; in the United States, for example, it was estimated that medication non-adherence costs approximately 100 billion dollars annually [9,10]. Research also found that medication non-adherence is associated with higher total healthcare costs and hospitalization costs among patients with type 2 diabetes mellitus (T2DM), suggesting the potential of bringing significant economic consequences at individual level [11]. In addition, medication non-adherence is also associated with higher risks for all-cause hospitalization and all-cause mortality in patients with DM [12]. Non-adherent patients with DM are also at greater risk of developing complications such as cardiovascular disease, and the diagnosis of complications is significantly related to a lower quality of life in patients with DM [13,14]. Though non-adherence to prescribed medications affects all patients regardless of their age-groups, older adults are at higher risk for non-adherence due to cognitive and functional impairments and prevalence of multiple comorbidities and medications, while they are more vulnerable to subsequent adverse health outcomes resulting from non-adherence due to age-related changes in pharmacokinetics and pharmacodynamics [15,16].

Given the significant health and economic consequences caused by medication non-adherence, especially among older adults with DM, it is imperative to detect non-adherent patients and identify barriers to medication adherence, so as to prevent and control the prevalence and costs of this diagnosable and treatable epidemic from further expanding [10]. Among various instruments available to determine behaviors of interest and measure medication non-adherence, the Morisky–Green–Levine (MGL) Medication Adherence Scale is one of the representative tools commonly used to diagnose medication non-adherence across various settings [10]. With regard to barriers to medication adherence, the World Health Organization (WHO) acknowledged that adherence to long-term therapies is a multidimensional phenomenon interactively influenced by five dimensions of factors, which are ‘social and economic factors, healthcare team and system-related factors, condition-related factors, therapy-related factors, and patient-related factors’ [16]. As the world population is aging faster than ever before with a rapidly increasing number of patients with DM, how to improve medication non-adherence in older adults with DM emerges as a potential challenge to the health systems of many countries, especially those with restrained resources, due to the call for systematic and multifaceted interventions [16,17,18].

With both the largest population with DM and the largest older population aged 60 years or above in the world, China, compared with many other countries, is at a higher risk of bearing heavy disease and economic burdens resulting from medication non-adherence in older patients with DM [17,18]. Though medication non-adherence in this vulnerable population group has received some attention in recent years, previous studies were mainly conducted at a single or a few hospitals or community health centers, which could not fully represent general older adults with DM due to the potential sampling bias and did not reflect the dichotomous rural-urban structure in China [19,20,21]. Furthermore, many only explored certain dimensions of factors associated with medication adherence without using a theoretically grounded conceptual framework and/or a commonly accepted medication adherence scale [19,20,21]. To our knowledge, little is known about how various dimensions of factors are simultaneously associated with medication non-adherence in older adults with DM in China. Filling this gap in the literature, this study aims to examine the extent of self-reported medication non-adherence in older adults with DM and to identify factors associated with this phenomenon.

## 2. Materials and Methods

### 2.1. Study Design and Data Collection

This study was based on the Shandong Province Elderly Family Health Service Survey conducted in 2017. Detailed sampling design has been described in previous publications [22]. In general, stratified multi-stage random sampling design was employed to interview older adults aged 60 or above, and the survey was administered in person. Caregivers did not complete the survey on behalf of respondents. In the first stage, a total of 6 county-level areas were selected as the primary sampling units (PSUs) out of 137 county-level areas in Shandong province, among which 1 urban district and 1 rural county were selected in each of eastern, central, and western regions of Shandong Province to reflect the regional socioeconomic disparities and the dichotomous rural-urban structure. In the second stage, 18 rural villages and 18 urban/suburban communities were selected from each PSU as the secondary sampling units (SSUs). In the third stage, an average number of 66 older adults were randomly selected per SSU based on rosters of local residents.

Among 7088 older adults who were selected and interviewed, 18 failed to complete the survey, resulting in a total sample of 7070 respondents. This study targeted a sample of 1002 older adults with DM. Respondents were identified as patients with DM if they were previously confirmed by physicians and were taking anti-diabetic medication/had taken anti-diabetic medication before. Respondents were perceived as having DM-related complication(s) if they received previous confirmation from physicians. Data with less than 10% of missing values were imputed with medians for continuous variables and modes for categorical or dichotomous variables. Before administering face-to-face interviews, informed consent for data collection and analysis was obtained from eligible participants who were willing and able to understand and answer interviewers’ questions.

### 2.2. Dependent Variable

We used the MGL Medication Adherence Scale that was proved to have good concurrent and predictive validity and good internal consistency [23]. Each of the four questions in the scale is asked in the reverse direction, the purpose of which is to reduce the potential bias of social desirability, namely, patients’ behaviors to please the interviewers to demonstrate that they are always taking their medications as instructed [23]. In the MGL Medication Adherence Scale, 0 point is assigned to the “yes” response, and 1 point is assigned to the “no” response [23]. Thus, the higher the score, the higher the adherence of the patients. The MGL Medication Adherence Scale assesses both unintentional medication non-adherence (forgetfulness or carelessness) and intentional medication non-adherence (stop taking prescribed medicine(s) when feeling better or worse) [23]. The full MGL score ranges from 0–4, with 0–1 being classified as having low adherence, 2–3 being classified as having moderate adherence, and 4 being classified as having high adherence [23]. Since the purpose of this study is to identify factors associated with medication non-adherence among older adults with DM, the full score was dichotomized into two groups: the adherent group (MGL score: 2–4) and the non-adherent group (MGL score: 0–1).

### 2.3. Independent Variables

Independent variables were chosen from the survey questionnaire according to the five dimensions of adherence conceptual framework [16]. Social and economic factors included gender (male, female), residence (urban, township, rural), age group (60–69, 70–79, ≥80), education level (junior or above, below junior), marital status (married, single), employment status (employed, unemployed), and personal annual income (USD >8882.7, USD 0–8882.7; USD 1 = CNY 6.7547 on average in 2017). Healthcare team and system-related factors included type of health insurance (Urban Employee Basic Medical Insurance (UEBMI), Urban and Rural Residents Basic Medical Insurance (URRBMI), others/none). Condition-related factors included disease duration (<5 years, ≥5 years) and incidence of complication (no, yes). Therapy-related factors included type of medication (oral hypoglycemic agent (OHA), insulin, both). Patient-related factors included perceived importance of medication adherence (important, unimportant) and self-rated mental health status in the past month (good, normal, poor).

### 2.4. Statistical Analysis

All analyses were conducted using Stata 16.1 (Stata Corp., College Station, TX, USA). Frequencies and percentages were calculated to describe categorical or dichotomous variables. We used chi-square test to determine the bivariate association between respondents’ characteristics and medication non-adherence. Unadjusted and adjusted logistic regression analyses were conducted to identify factors influencing medication non-adherence among older adults with DM. Independent variables with a *p*-value of less than 0.2 in the unadjusted analyses were selected as candidate independent variables for the adjusted model. We employed backward elimination on candidate independent variables with a *p*-value cutoff of 0.05 to identify independent variables significantly associated with medication non-adherence. Strength of associations between respondents’ characteristics and medication non-adherence was described using crude odds ratios (CORs) and adjusted odds ratios (AORs) with the corresponding 95% confidence intervals (CIs).

### 2.5. Ethical Considerations

The Research Ethics Committee of Shandong University granted the ethical approval for this survey (No. 20170110). Participation in the interview was voluntary with informed consent, and respondents’ data were collected anonymously.

## 3. Results

### 3.1. Respondents’ Characteristics by the Level of Medication Adherence

Table 1 shows respondents’ characteristics by the level of medication adherence. Of a total of 1002 older adults with DM, the majority of respondents were female (69.6%), aged under 80 years old (95.9%), received an education below junior level (71.5%), married (82.9%), unemployed (77.5%) and earned a personal annual income of USD 8882.7 or below (96.8%). As for other dimensions of characteristics, 70.6% (*n* = 707) had URRBMS, 67.4% (*n* = 675) had a disease duration of 5 years or above, 84.1% (*n* = 843) did not experience the incidence of complication, 84.4% only took OHA (*n* = 846), 90.6% (*n* = 908) perceived medication adherence to be important, and 75.4% (*n* = 756) had good mental health status. The overall prevalence of medication non-adherence was 19.9%, and the gender-specific prevalence was 15.4% in males and 21.8% in females.

### 3.2. Respondents’ Self-Reported Medication Non-Adherence by the MGL Medication Adherence Scale

Table 2 presents the details of respondents’ self-reported medication non-adherence by the MGL Medication Adherence Scale. Among 1002 respondents, 421 (42.0%) sometimes forgot to take prescribed medicine, 299 (29.8%) were careless occasionally about taking medicine; 254 (25.4%) sometimes stopped taking medicine when feeling better; and 170 (17.0%) stopped taking medicine if they felt worse when taking the medicine sometimes. Out of 1002 respondents, 46.2% (*n* = 463) were highly adherent, 33.9% (*n* = 340) had moderate adherence, and 19.9% (*n* = 199) had low adherence. In addition, the percentage of respondents reporting unintentional non-adherence was 44.5% (*n* = 446), the percentage of respondents reporting intentional non-adherence was 33.2% (*n* = 333), and the percentage of respondents reporting both unintentional and intentional non-adherence was 24.0% (*n* = 240).

### 3.3. Factors Influencing Medication Non-Adherence among Older Adults with DM

The unadjusted and adjusted associations between respondents’ characteristics and medication non-adherence are presented in Table 3. In the unadjusted model, female respondents (COR = 1.53, 95% CI: 1.07–2.19), those who perceived medication adherence to be unimportant (COR = 1.72, 95% CI: 1.07–2.78) and self-reported normal mental status (COR = 1.48, 95% CI: 1.01–2.16) were more likely to self-report medication non-adherence. Respondents aged 70–79 years old (COR = 0.69, 95% CI: 0.50–0.95) and those with longer disease duration (COR = 0.64, 95% CI: 0.47–0.89) were less likely to self-report medication non-adherence. In the adjusted model, several factors were still independently associated with medication non-adherence after controlling for other factors. In particular, female participants were more likely than male participants to experience medication non-adherence (AOR = 1.56, 95% CI: 1.09–2.24). Respondents who have disease durations of 5 years or longer were less likely to experience medication non-adherence than respondents with shorter disease duration (AOR = 0.63, 95% CI: 0.46–0.87). Respondents who perceived medication adherence to be unimportant were more likely to experience medication non-adherence than those who perceived medication non-adherence as important (AOR = 1.69, 95% CI: 1.05–2.74).

## 4. Discussion

Medication non-adherence is a serious challenge to the self-management of DM among adults with DM, especially among older adults, and its downstream effects will be multiplied if left unaddressed and will be manifested as increased incidence and prevalence of major complication and heavier disease and economic burdens of the disease. Therefore, the objectives of this study were to examine the prevalence of medication non-adherence among older adults with DM and to identify its influencing factors.

The prevalence of self-reported medication non-adherence among older adults with DM in Shandong province was 19.9% in this study, which was close to the finding reported from Cambodia (17.2%) [24] but lower than the finding reported from Algeria (31.3%) [25]. Disparities between findings could be explained by differences in the sampling design, study settings, health systems, respondents’ socioeconomic characteristics, and/or instruments used to measure medication adherence.

In this current study, females were more likely to be non-adherent to their prescribed medications than males, which is consistent with some previous studies [26,27]. Considering that there is mixed evidence for the association between medication non-adherence and gender [25,28], future research is needed to explore the mechanisms behind gender effects. We found that disease duration was an independent factor influencing medication non-adherence. The longer the disease duration, the smaller the likelihood of older adults with DM being non-adherent to their prescribed medications. A possible explanation for this association is that patients gain increasing knowledge and awareness of DM as the disease progresses, which lays the foundation for a better adherence to prescribed medications [29].

Our study also demonstrated that respondents who perceived medication adherence as unimportant were more likely to experience medication non-adherence. This finding could be potentially attributed to the property of the MGL Medication Adherence Scale that it measures both intentional non-adherence (forgetfulness or carelessness) and un-intentional medication non-adherence (stop taking prescribed medication(s) when feeling better or worse) [23]. In our study, the respondents in the non-adherent group had an MGL score of 0–1, which implies that non-adherent respondents, regardless of which specific scale items to which they answered “yes”, experienced both intentional and un-intentional medication adherence. Therefore, it is intuitive that perceived importance of medication adherence was statistically significantly associated with medication non-adherence among older adults with DM. Fortunately, this factor is modifiable through providing counseling and health education programs, which demonstrated promising effects in many settings [30,31]. To overcome this barrier to adherence, health professionals will play a central role in changing patients’ perception of the importance of medication adherence and improving self-management of DM.

This study has several strengths and limitations. The stratified multistage random sampling design of this study ensured that enrolled respondents represented different socioeconomic backgrounds. Additionally, the sample size of this study was larger compared with many previous studies and therefore is of better potential to identify factors associated with medication non-adherence. Furthermore, this study targeted older adults with DM, a population group that is vulnerable to the negative consequences of medication non-adherence, and provided evidence on the characteristics of older patients who were at higher risks of experiencing medication non-adherence.

Several limitations of the present study should also be noted while interpreting the findings. First, this study was based on patients’ self-reported medication adherence and therefore was inevitably subject to recall bias and self-report bias. Second, the study did not distinguish between patients with type 1 diabetes mellitus (T1DM) and patients with type 2 diabetes mellitus (T2DM), nor did it distinguish between different types of DM complications. Third, the cross-sectional design of this study only allows for the establishment of association between respondents’ characteristics and medication adherence. Finally, this study only considered factors available in the survey questionnaire, and we acknowledge that there are some other confounding factors associated with medication non-adherence in addition to the factors included in this study. To better understand factors influencing medication non-adherence among older adults with DM and explore underlying mechanisms, future study need to pay more attention to the collection of DM-specific data, such as types of complications and types and doses of anti-diabetic medications.

## 5. Conclusions

In conclusion, this study showed that about one out of five older adults with DM in Shandong province, China, experienced medication non-adherence, and that gender, disease duration and perceived importance of medication adherence were associated with medication non-adherence in this population group. Medication non-adherence among Chinese older adults with DM is a public health problem that demands attention and efforts from decision-makers and professionals in the healthcare sector. To counter this pressing challenge, future priorities could be to provide counseling and health education programs to raise patients’ awareness of the importance of medication adherence and improve patients’ self-management of DM.

## Figures and Tables

**Table 1:** Summary of respondents’ characteristics by level of medication adherence.

Characteristics	Total	Adherent	Non-Adherent	χ^2^	*p*-Value
N	1002	803 (80.1)	199 (19.9)		
***Social and economic factors***					
Gender				5.456	0.019
Male	305 (30.4)	258 (84.6)	47 (15.4)		
Female	697 (69.6)	545 (78.2)	152 (21.8)		
Residence				4.527	0.104
Urban	326 (32.5)	273 (59.4)	53 (64.0)		
Township	72 (7.2)	54 (6.7)	18 (9.1)		
Rural	604 (60.3)	476 (33.9)	128 (26.9)		
Age group				5.224	0.073
60–69	528 (52.7)	409 (77.5)	119 (22.5)		
70–79	433 (43.2)	361 (83.4)	72 (16.6)		
≥80	41 (4.1)	33 (80.5)	8 (19.5)		
Education level ^a^				2.953	0.086
Junior or above	286 (28.5)	239 (83.6)	47 (16.4)		
Below junior	716 (71.5)	564 (78.8)	152 (21.2)		
Marital status				2.863	0.091
Married	831 (82.9)	674 (81.1)	157 (18.9)		
Single ^b^	171 (17.1)	129 (75.4)	42 (24.6)		
Employment status				1.927	0.165
Employed	225 (22.5)	173 (76.9)	52 (23.1)		
Unemployed ^c^	777 (77.5)	630 (81.1)	147 (18.9)		
Personal Annual Income				3.850	0.050
USD >8882.7	32 (3.2)	30 (93.8)	2 (6.2)		
USD 0–8882.7	970 (96.8)	773 (80.0)	197 (20.0)		
***Health care team and system-related factors***					
Health insurance ^d^				3.469	0.176
UEBMS	262 (26.1)	220 (84.0)	42 (16.0)		
URRBMS	707 (70.6)	556 (78.6)	151 (21.4)		
Others/None	33 (3.3)	27 (81.8)	6 (18.2)		
***Condition-related factors***					
Disease duration				7.354	0.007
<5 years	327 (32.6)	246 (75.2)	81 (24.8)		
≥5 years	675 (67.4)	557 (82.5)	118 (17.5)		
Complication				0.312	0.576
No	843 (84.1)	673 (79.8)	170 (20.2)		
Yes	159 (15.9)	130 (81.8)	29 (18.2)		
***Therapy-related factors***					
Type of medication				3.323	0.190
OHA ^e^	846 (84.4)	670 (79.2)	176 (86.6)		
Insulin	70 (7.0)	61 (8.1)	9 (5.2)		
Both	86 (8.6)	72 (9.6)	14 (8.1)		
***Patient-related factors***					
Perceived importance of medication adherence				5.120	0.024
Important	908 (90.6)	736 (81.1)	172 (18.9)		
Unimportant	94 (9.4)	67 (71.3)	27 (28.7)		
Self-rated mental health status				4.080	0.130
Good	756 (75.4)	616 (81.5)	140 (18.5)		
Normal	183 (18.3)	137 (74.9)	46 (25.1)		
Poor	63 (6.3)	50 (79.4)	13 (20.6)		

^a^ Junior or above: middle school/high school/technical school/technical secondary school/junior college/undergraduate or above; below junior: illiterate/elementary school; ^b^ single: single/divorced/widowed/others; ^c^ unemployed: retired/unemployed; ^d^ Urban Employee Basic Medical Insurance (UEBMI) and Urban and Rural Residents Basic Medical Insurance (URRBMI) are government-run health insurance programs launched for different population groups with an aim of achieving universal coverage of the Chinese population and health services; ^e^ oral hypoglycemic agent (OHA).

**Table 2:** Details of respondents’ self-reported medication non-adherence status.

**Item of the MGL Medication Adherence Scale ^a^**	**Frequency (Yes)**	**Percentage (%)**
Do you ever forget to take your medicine?	421	42.0
Are you careless at times about taking your medicine?	299	29.8
When you feel better, do you sometimes stop taking your medicine?	254	25.4
Sometimes if you feel worse when you take the medicine, do you stop taking it?	170	17.0
**Level of Medication Adherence**	**Frequency**	**Proportion (%)**
High adherence	463	46.2
Moderate adherence	340	33.9
Low adherence	199	19.9
**Intentional versus Unintentional Non-adherence**	**Frequency**	**Percentage (%)**
Unintentional non-adherence	446	44.5
Intentional non-adherence	333	33.2
Both	240	24.0
Total	1002	100.0

^a^ the Morisky–Green–Levine (MGL) Medication Adherence Scale.

**Table 3:** Factors influencing medication non-adherence among older adults with DM.

Characteristics	Level	Unadjusted Model		Adjusted Model ^a^	
(Reference Group)		COR ^b^ (95% CI)	*p*-Value	AOR ^c^ (95% CI)	*p*-Value
Gender (Male)	Female	1.53 (1.07–2.19)	0.020	1.56 (1.09–2.24)	0.016
Residence (Urban)	Township	1.72 (0.93–3.16)	0.082		
	Rural	1.39 (0.97–1.97)	0.070		
Age group (60–69)	70–79	0.69 (0.50–0.95)	0.023		
	≥80	0.83 (0.37–1.85)	0.654		
Education level (Junior or above)	Below junior	0.90 (0.79–1.03)	0.113		
Marital status (Married)	Single	1.40 (0.95–2.06)	0.092		
Employment status (Employed)	Unemployed	0.78 (0.54–1.11)	0.166		
Personal annual income (USD >8882.7)	USD 0–8882.7	3.82 (0.91–16.13)	0.068		
Insurance (UEBMI)	URRBMI	1.42 (0.98–2.07)	0.066		
	Others/None	1.16 (0.45–2.99)	0.753		
Disease duration (<5 years)	≥5 years	0.64 (0.47–0.89)	0.007	0.63 (0.46–0.87)	0.005
Complication (No)	Yes	0.88 (0.57–1.37)	0.577		
Type of medication (OHA)	Insulin	0.56 (0.27–1.15)	0.116		
	Both	0.74 (0.41–1.34)	0.323		
Perceived importance of adherence (Important)	Unimportant	1.72 (1.07–2.78)	0.025	1.69 (1.05–2.74)	0.032
Mental health status (Good)	Normal	1.48 (1.01–2.16)	0.045		
	Poor	1.14 (0.60–2.16)	0.679		

^a^ Independent variables were considered for the adjusted model if they had a *p*-value of 0.2 or less in the unadjusted model; ^b^ crude odds ratio (COR); ^c^ adjusted odds ratios (AOR).

## Abbreviations

DM	Diabetes Mellitus
T2DM	Type 2 Diabetes Mellitus
MGL	Morisky–Green–Levine Medication Adherence Scale
UEBMI	Urban Employee Basic Medical Insurance
URRBMI	Urban and Rural Residents Basic Medical Insurance
OHA	Oral Hypoglycemic Agent
COR	Crude Odds Ratio
AOR	Adjusted Odds Ratio
